# Impacts of Drought on Photosynthesis in Major Food Crops and the Related Mechanisms of Plant Responses to Drought

**DOI:** 10.3390/plants13131808

**Published:** 2024-06-30

**Authors:** Meiyu Qiao, Conghao Hong, Yongjuan Jiao, Sijia Hou, Hongbo Gao

**Affiliations:** National Engineering Research Center for Tree Breeding and Ecological Restoration, State Key Laboratory of Efficient Production of Forest Resources, College of Biological Sciences and Biotechnology, Beijing Forestry University, Beijing 100083, China; qiaomeiyu123@163.com (M.Q.);

**Keywords:** drought, stress, photosynthesis, chloroplast, crop

## Abstract

Drought stress is one of the most critical threats to crop productivity and global food security. This review addresses the multiple effects of drought on the process of photosynthesis in major food crops. Affecting both light-dependent and light-independent reactions, drought leads to severe damage to photosystems and blocks the electron transport chain. Plants face a CO_2_ shortage provoked by stomatal closure, which triggers photorespiration; not only does it reduce carbon fixation efficiency, but it also causes lower overall photosynthetic output. Drought-induced oxidative stress generates reactive oxygen species (ROS) that damage cellular structures, including chloroplasts, further impairing photosynthetic productivity. Plants have evolved a variety of adaptive strategies to alleviate these effects. Non-photochemical quenching (NPQ) mechanisms help dissipate excess light energy as heat, protecting the photosynthetic apparatus under drought conditions. Alternative electron pathways, such as cyclical electron transmission and chloroplast respiration, maintain energy balance and prevent over-reduction of the electron transport chain. Hormones, especially abscisic acid (ABA), ethylene, and cytokinin, modulate stomatal conductance, chlorophyll content, and osmotic adjustment, further increasing the tolerance to drought. Structural adjustments, such as leaf reordering and altered root architecture, also strengthen tolerance. Understanding these complex interactions and adaptive strategies is essential for developing drought-resistant crop varieties and ensuring agricultural sustainability.

## 1. Introduction

Drought, which refers to a period of water deficit, negatively affects growth rate and productivity. This phenomenon is of utmost importance in agriculture because a lack of water significantly lowers crop yield and quality. Drought stress occurs when the amount of water reaching the plant roots may not be enough for the transpiration of the leaves, leading to a reduction in water potential and cell dehydration [1]. Drought stress is a challenging and debilitating factor that limits agricultural productivity, adversely affecting crop quality and yield while exacerbating food shortages. It is expected that the intensity and frequency of drought stress will increase as a result of global climate change, insufficient food, water scarcity, and increased population. Drought has been reported to influence various physiological and biochemical processes such as photosynthesis, nitrogen assimilation, and metabolism [2,3,4,5,6]. Plants undergo several physiological modifications during drought to maximize water use while maintaining essential pathways [3,6,7,8,9,10]. Some of these physiological behaviors include stomatal closure to minimize water loss via transpiration and root system changes aimed at enhancing water uptake [4,7]. Plants also accumulate neutral solutes such as sugars, proline, and sugar alcohols, which stabilize proteins and cellular structures [11,12]. Although their major objective is to aid in drought recovery, the physiological responses can affect growth and yield, necessitating a critical assessment of drought stress in plants [13]. The goal is to understand drought resistance and breed a variety of plants tolerant to this need. Plant physiologists and biologists have made significant contributions to unraveling drought-responsive genes and pathways that could be modified to protect plants against drought challenges [6,14,15,16]. Due to limitation of space, this review will mainly focus on major crops such as rice and maize.

### Drought Leads to Significant Yield Losses in Major Food Crops

In 2023–2024, the yield data for major crops across several countries (regions) highlight the yield and significance of staple foods such as maize, rice, soybean and wheat (Table 1). In China, maize (*Zea mays*) achieved a yield of 6.5 tons per hectare (T/Ha) over an area of 44,218 thousand hectares (Ha), while rice (*Oryza sativa*) and wheat (*Triticum aestivum*) had yields of 7.1 T/Ha and 5.8 T/Ha, respectively (Table 1). These data underscore the reliance on these crops for food security and highlight the potential impacts of environmental stresses such as drought on their productivity. Drought stress affects crop yield drastically, significantly reducing the productivity of major food crops such as wheat, rice, and maize. Wheat, rice, and maize are staples that contribute much of the caloric content in the human diet. Drought conditions affect many physiological processes in crops, leading to reduced growth, development, and eventual yields [17].

Rice responds significantly to drought stress, especially during the reproductive stages. Drought stress at flowering results in a significant reduction in the number of filled grains, causing a decrease in grain yield. This effect occurs mainly because of poor development of pollen and reduced pollen viability, leading to a lower seed set [18]. Drought also affects rice photosynthesis and respiration through stomatal closure and reduced carbon assimilation. In addition, there is a limitation in the availability of water for the translocation of assimilates to the grain, causing reduced grain yield. Research shows that severe levels of drought stress can cause up to a 50% reduction in rice yield, making the resistance of rice varieties to drought a priority [19].

Maize is also a staple food crop internationally impacted by drought stress. Drought has a massive impact on maize yield, especially when it occurs during flowering and grain filling. Drought stress during flowering causes a reduction in the number of viable kernels due to poor silk receptivity and pollen shed, which increases kernel set, causing reduced yield [20]. Similarly, drought during the grain filling stage limits assimilates due to low photosynthetic activity and nutrient uptake. This results in smaller kernel size and kernel weight, causing further yield reduction. Drought stress research indicates that reduced yields can reach up to 40% depending on the severity and stage of the crop [21]. The impact of these percentages is significant for maize, as it is central to food and feed in countries that heavily rely on maize consumption and production.

For dryland crops such as wheat, drought mostly affects the grain fill period, causing smaller grain sizes and grain weight. Grain yield reduction occurs due to decreased photosynthesis, nutrient uptake, and accelerated leaf senescence, which reduces carbohydrate production and translocation to the developing grains [22] Drought stress during the flowering period can cause sterility and a significant reduction in grain number and eventual yield [23].

## 2. Impacts of Drought on Photosynthesis

### 2.1. Drought Affects Light-Dependent Reactions of Photosynthesis

Drought stress has a significant impact on the light-dependent reactions of photosynthesis, which account for the majority of photosynthetic efficiency. Light reactions take place in the thylakoid membranes of chloroplasts and utilize light energy absorbed by chlorophyll to generate ATP and NADPH via photosystems II (PS II) and I (PS I), respectively. Since light reactions are highly dependent on water, drought often substantially affects these reactions. Impairment of light reactions leads to various undesirable effects that harm plant productivity, making it a critical aspect to study.

**Figure 1:**
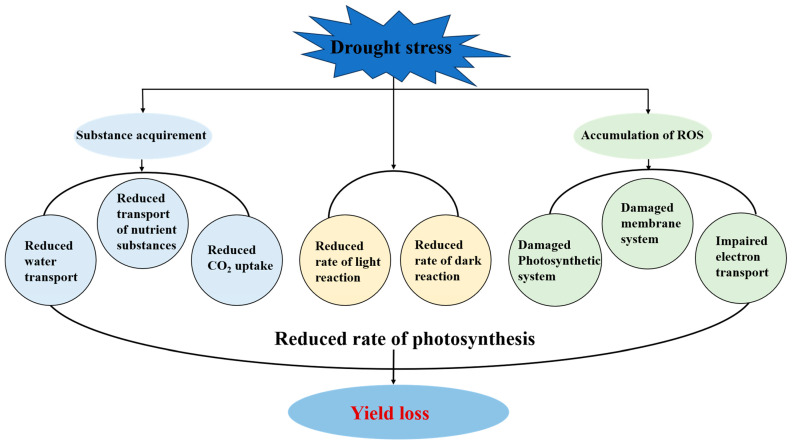
Effects of drought stress on the photosynthesis of major food crops. Drought stress not only reduces the rate of light reaction and dark reaction, but also restricts the acquirement of the substances for photosynthesis. Moreover, drought stress results in the accumulation of reactive oxygen species (ROS), which causes various damages in chloroplasts.

Firstly, drought stress specifically affects PS II due to its extreme susceptibility to water deficit. PSII loses its stability and ability to perform under drought conditions (Figure 1), resulting in a substantial decrease in the efficiency of light energy conversion. As part of the photosynthetic electron transport chain, the D1 protein of PSII is prone to oxidative stress and damage from ROS generated under drought, which disrupts water splitting [24]. This, in turn, decreases oxygen evolution and restricts electron transfer from water to the plastoquinone pool, reducing photochemical efficiency and making it vulnerable to photoinhibition and photodamage [25].

Unlike PSII, PSI is generally unaffected by drought, although the electron flow between photosystems is reduced due to PSII impairment. Consequently, the redox state of the electron transport chain is unbalanced, resulting in an over-reduction of PSI electron acceptors and excessive ROS generation, which further harm the photosynthetic machinery [26].

The final critical aspect of drought-induced light reaction harm is the inhibition of linear electron flow (Figure 1). Under normal conditions, linear electron transport transfers electrons from water to NADP^+^ utilizing PSII, the cytochrome *b*_6_*f* complex, PSI, and a proton gradient across the thylakoid membrane. Drought harms these components and increases resistance within the gradient, thus also increasing the membrane’s excitation energy content. This can damage PSI and PSII via photodamage and photosynthetic inactivation if not dissipated safely, decreasing productivity [27].

### 2.2. The Impact of Drought on Dark Reactions

Dark reactions of photosynthesis, more specifically the Calvin cycle, are also significantly affected by drought stress (Figure 1). The Calvin cycle is a series of reactions that occur in the stroma of chloroplasts and serve to fix carbon and synthesize sugars using products of the light reactions, ATP and NADPH. Drought conditions disrupt the Calvin cycle in multiple ways, which involve a decrease in the activity of enzymes, reduced carbon fixation, and difficulties in the export of photosynthetic products from the chloroplasts to the cytoplasm.

One of the main effects of drought on the Calvin cycle is the decreased activity of enzymes. Ribulose-1,5-bisphosphate carboxylase/oxygenase (Rubisco), the enzyme catalyzing the first reaction of carbon fixation, is critical to the Calvin cycle. Rubisco is very sensitive to drought, and stress conditions reduce its carboxylation efficiency. This is due to the reduced availability of the substrates, ribulose-1,5-bisphosphate (RuBP), and CO_2_ [28]. Stomatal conductance is reduced under drought, which reduces CO_2_ uptake, and decreases the internal CO_2_ concentration and inhibits the enzyme. Drought also causes oxidative stress, affecting the synthesis and activation of Rubisco and limiting its carboxylation activity [29].

Rubisco activase is another key enzyme affected by drought stress. Its thermal lability and sensitivity to drought lead to the inhibition of Rubisco activase, which limits Rubisco’s ability to supply the cycle with substrates by promoting the removal of inhibitory sugar phosphates [30]. In addition, the activity of other enzymes involved in the Calvin cycle is lower. Phosphoribulokinase (PRK) and glyceraldehyde-3-phosphate dehydrogenase (GAPDH), the enzymes involved in the regeneration of RuBP and the reduction of 3-phosphoglycerate to glyceraldehyde-3-phosphate, have lower activity under drought [31].

Drought stress also disrupts the export of photosynthetic products from the chloroplast to the cytoplasm. The synthesis of triose phosphates, such as glyceraldehyde-3-phosphate and dihydroxyacetone phosphate, is reduced under drought conditions due to the limited availability of ATP and NADPH. These triose phosphates are essential intermediates that need to be exported to the cytoplasm for further metabolism and storage [28]. The reduced export of triose phosphates leads to an accumulation of these intermediates in the chloroplast, which can feedback-inhibit the Calvin cycle and exacerbate the reduction in carbon fixation efficiency.

Furthermore, the impaired export of photosynthetic products affects the overall metabolic balance within the plant. The reduced availability of sugars in the cytoplasm limits the synthesis of sucrose, starch, and other carbohydrates, which are crucial for growth and development. This metabolic imbalance leads to a decrease in biomass accumulation and affects various physiological processes, including cell wall synthesis, energy storage, and stress responses [32,33]. The impaired export of photosynthetic products also affects the signaling pathways that regulate the plant’s response to drought stress, further compromising the plant’s ability to adapt to water deficit conditions [34].

## 3. Other Impacts of Drought on Major Food Crops

### 3.1. Reactive Oxygen Species (ROS) Production

Drought stress induces the production of ROS in plants, which leads to extensive cellular damage and impairs photosystems (Figure 1). As summarized above, one of the primary targets of ROS is the photosynthetic apparatus, such as PSII and the electron transport chain. Additionally, ROS can damage the lipids in thylakoid membranes through lipid peroxidation, leading to membrane destabilization and impaired photosynthetic efficiency. The integrity of the chloroplast membranes is critical for maintaining the proper function of the photosynthetic machinery, and any disruption can severely affect the plant’s photosynthesis [35].

Beyond the photosystems, the oxidative stress induced by drought also affects nucleic acids, leading to mutations, strand breaks, and the formation of adducts. These damages can interfere with the replication and transcription processes, affecting the overall cellular metabolism and stress response mechanisms [36]. Moreover, the accumulation of ROS under drought conditions can lead to the activation of programmed cell death (PCD) pathways, resulting in the loss of cells and tissues critical for plant growth and development. The interplay between ROS production, antioxidant defenses, and cellular repair mechanisms determines the extent of damage and the plant’s ability to recover from drought-induced oxidative stress [35].

### 3.2. Drought Affects the Channels for Substance Transport

Drought stress has a severe impact on the transport of nutrients and water in plant tissues (Figure 1), disrupting the normal function of the phloem and xylem. These vascular systems are essential for the regulation of plant hydration and nutrient distribution and the maintenance of physiological balance. The efficiency of the phloem and xylem is affected by drought, which causes several detrimental effects on plant growth and productivity.

The xylem is responsible for the upward transpiration stream of water and dissolved minerals from the root to aerial parts of a plant. The process of water transport in the xylem is facilitated by a cohesive tension mechanism that requires a continuous water column. Drought conditions lead to soil water deficits, which reduce the availability of water for uptake by roots, lowering the water potential gradient between soil and roots. Consequently, the movement of water into a plant during drought stress is hindered. As drought stress becomes more severe, the cohesion of water columns may be broken, causing cavitation and embolism. Cavitation arises when there are air bubbles in xylem vessels, which disrupt the continuity of the water column, making water transport inefficient. This process significantly affects the moisture level in plants, reducing their ability to maintain turgor pressure. Wilting occurs when plants cannot maintain their turgor, with a reduction in photosynthesis accompanying it [37].

In addition to cavitation, drought instigates the deposition of callose in the xylem vessels. Callose is a polysaccharide that occludes the xylem vessels, thus limiting water movement. This response is protective as it limits injury and the spread of air emboli, but it also increases water limitation. This reflects the complexity of plant responses to water scarcity and the balance between defensive function and activity limitation [38].

Phloem transport, responsible for the distribution of assimilates including photosynthates and other organic compounds from source to sink tissues, may also be compromised. Phloem transport depends on turgor pressure due to the osmotic potential of solutes to facilitate the flow of assimilates. Under drought, reduced water availability also reduces phloem turgor pressure, leading to impaired transport of nutrients and signaling molecules. The distribution of essential nutrients including carbohydrates and amino acids is compromised with callose deposition in the sieve plates, limiting the flow of assimilates into the sink tissues [39].

Plasmodesmata, microchannels responsible for substance transport between plant cells, also experience callose deposition to regulate cell-to-cell transport under stress conditions. Functional impairment may occur if excess callose deposition occludes the transport channels [40]. Additionally, nutrient transport is disrupted, limiting the synthesis of essential compounds used for physiological processes. Nitrogen, phosphorus, and potassium are macronutrients required for enzyme activation, osmotic regulation, and photosynthesis, among other processes. Their low uptake and transport due to drought stress leads to deficiencies affecting cellular metabolism. The low availability of such nutrients deteriorates the condition of a plant under drought conditions, leading to slow growth, low yield, and reduced resistance to other stresses [41].

Sucrose is synthesized in mesophyll cells, then moves from source organ cells into sink organ cells via a phloem loading system to support plant growth and development [32]. In addition to plasmodesmata, two membrane proteins, SUT (Sucrose Transporter) and SWEET (Sugars Will Eventually be Exported Transporters)-type transporters, participate in apoplastic loading between companion cells and the sieve element [42,43,44]. In rice, under drought, the transcript levels of *OsSUT4* and *OsSUT5* were reduced at the vegetative stage [45].

## 4. Mechanisms of Photosynthetic Response to Drought

### 4.1. Non-Photochemical Quenching Mechanisms

**Figure 2:**
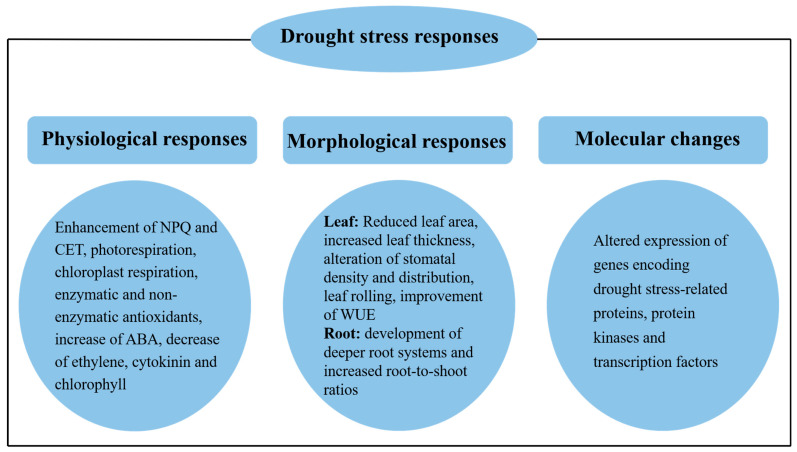
Drought stress responses in major food crops, with an emphasis on photosynthesis. Plants respond to drought at various levels to protect photosynthesis and other aspects of plants. NPQ, non-photochemical quenching. CET, cyclic electron transfer. WUE, water use efficiency.

Non-photochemical quenching (NPQ) mechanisms are indeed essential for protecting plants from the harmful effects of excess light, especially under drought stress (Figure 2). Non-photochemical quenching is triggered by the transthylakoid proton gradient and a set of processes involving mechanisms responsible for the dissipation of excess excitation energy to generate heat and prevent overexcitation of chlorophyll molecules, leading to the photodamage of PSII [46,47,48]. These mechanisms are essential in maintaining photosynthesis and protecting the photosystem during water stress.

In rice, NPQ is very essential in alleviating the deleterious effects of drought-induced oxidative stress. Drought causes a serious reduction in CO_2_ availability due to the closure of stomata, leading to the over-reduction of the electron transport chain (ETC) and the accumulation of ROS. This mechanism protects the photosystem against photodamage. One of the components of NPQ is the xanthophyll cycle, which synthesizes zeaxanthin from violaxanthin. Zeaxanthin plays a crucial role in the dissipation of heat upon excess energy, reducing photo-oxidative damage [49]. Additionally, the PsbS protein is responsible for the activation of NPQ. This protein responds to changes in lumen pH by converting excess energy into heat, reducing the damage caused by drought [50,51].

The types of NPQ in rice include energy-dependent quenching (qE), state transition quenching (qT), and photoinhibitory quenching (qI). qE occurs at a low rate and responds to the xanthophyll cycle. qT involves the redistribution of light-harvesting complex II (LHCII) between PSII and PSI to balance the excitation energy distribution. qI is more sustained and responds to long-term exposure to high light [52].

In maize, similarly to rice, NPQ mechanisms are mostly based on the xanthophyll cycle. In maize, zeaxanthin helps the plant thermally dissipate heat, increasing the efficiency of heat dissipation. Like rice, maize has qE and qT. The regulation of NPQ in maize is elicited by the xanthophyll cycle and LHCII [53].

### 4.2. Enhancement of Alternative Electron Transfer

The integration of alternative electron transfer mecha5nisms such as cyclic electron transfer (CET) and chloroplast respiration is critical for maintaining photosynthetic performance and protecting photosystems under drought stress (Figure 2). Alternative pathways are involved in balancing energy and dissipating excess energy to prevent oxidative stress, thus ensuring that plants remain operational and survive in water-limited environments.

CET around PSI is a critical pathway for rice adaptation to drought stress. The CET process involves the flow of electrons from ferredoxin (Fd) back to the plastoquinone pool (PQ), ultimately returning to PSI. During this process, ATP is generated by the Fd-dependent NAD(P)H dehydrogenase (NDH) complex, whereas NADPH is not produced. The generated ATP maintains redox homeostasis through the maintenance of the ATP/NADPH ratio, which is essential for various metabolic pathways. Under drought conditions, the demand for ATP increases due to the need for active transport, osmotic adjustment, and active repair mechanisms. The CET channel prevents photo-damage produced by the electron transport chain and ROS that inhibit PSII through the modulation of electron flow [54].

Another mechanism that increases drought resilience is chloroplast respiration—more precisely, the activity of the chloroplast ATP synthase and the plastid terminal oxidase (PTOX). PTOX facilitates the oxidation of the plastoquinone pool during the transfer of electrons. Electrons are transferred from the plastoquinone pool to oxygen, forming a water molecule and preventing the production of singlet oxygen. The flow of electrons helps to prevent the over-reduction of the photosynthetic electron transport chain and the subsequent generation of ROS. This mechanism is particularly important under increased drought stress. Chloroplast respiration maintains photosynthetic efficiency and protects the photosystems from excess energy through an alternative sink of electrons. The integration of genes coding for PTOX enhances its effectiveness [55].

The same mechanisms are involved in maize to increase drought resilience. CET promotes the maintenance of the balance of ATP and NADPH under drought conditions. CET generates ATP, which is required for the stimulation of NPQ mechanisms. The process involves the dissipation of excess light energy as heat. Chloroplast respiration also supports the process through increased PTOX activity; the oxidase acts as an alternative sink of electrons. Such a process protects the photosystems from excess energy [56].

### 4.3. Photorespiration

**Figure 3:**
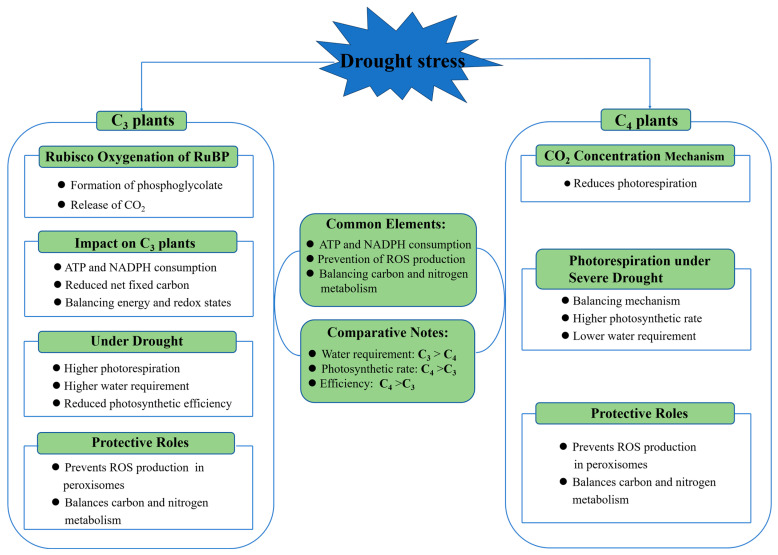
Photorespiration and drought response in C_3_ and C_4_ plants. The mechanisms of photorespiration and drought response in C_3_ (such as rice) and C_4_ (such as maize) plants differ. Under drought stress, both C_3_ and C_4_ plants increase ATP and NADPH consumption and reduce ROS production. There are higher water consumption requirements and increased photorespiration in C_3_ plants, while C_4_ plants exhibit higher photosynthetic rates and efficiency.

Photorespiration occurs in plants when Rubisco, ribulose-1,5-bisphosphate carboxylase/oxygenase, the all-important enzyme, oxygenates RuBP, ribulose-1,5-bisphosphate, instead of carboxylating it. This reaction leads to the formation of phosphoglycolate, which eventually releases CO_2_. Photorespiration is generally wasteful because it counteracts productivity in photosynthesis by reducing the net amount of fixed carbon and consuming ATP and NADPH without producing sugars. However, photorespiration also plays essential protective roles by consuming excess ATP and NADPH, preventing the over-reduction of energy and redox in the electron transport chain, and limiting ROS production in the peroxisome under certain stress conditions, including drought (Figure 3). Moreover, it generates vast sets of usable intermediates that can help plants be metabolically flexible and maintain metabolic resilience under drought [57].

Photorespiration is a predominant process in plants even under drought, particularly in C_3_ plants like rice and C_4_ plants like maize (Figure 3). Rice plants undergo more photorespiration than maize plants because Rubisco tends to oxygenate RuBP directly, primarily due to its energy consumption. While it is an energy-intensive process, it is invaluable as a safeguard mechanism that ensures that the energy and redox states are balanced, and produces metabolic intermediates used in stress responses [57].

Maize plants have evolved fewer photorespiration processes due to mechanisms to concentrate CO_2_ into the C_4_ carboxylating enzyme, but can also engage in photorespiration as a critical balancing metabolic event under severe drought (Figure 3). Maize plants under drought have a higher photosynthetic rate than rice plants due to the effectiveness of the C_4_ pathway in concentrating CO_2_ around the enzyme. Therefore, rice requires about fifty percent more water in creating a macromolecular system.

### 4.4. Plant Hormones and Chlorophyll Content

Plant hormones regulate the response of plants to drought stress [58], and the physiological processes that mediate drought tolerance are known to be affected by these hormones (Figure 2). Well-known plant hormones involved in abiotic stress include abscisic acid, ethylene, and cytokinins. Drought conditions affect chlorophyll arrangement, which is predominantly important to allow maximum light absorption and maintain photosynthesis (Figure 2).

Abscisic acid (ABA) is considered the central plant hormone for photosynthesis suppression during drought. In response to water deficits in rice, ABA levels increase within the tissues, inducing the expression of numerous defense responses. One of ABA’s primary functions is to trigger stomatal closure, reducing water loss. It binds to receptors in the guard cells, leading to the redistribution of membrane proteins, resulting in ion outflow and reduced turgor pressure in the guard cells. This reduction facilitates stomatal pore closure, resulting in minimal transpiration to reduce water loss, but concurrently restricts CO_2_ uptake, thus inactivating photosynthesis. Also, ABA promotes chlorophyll degradation under drought conditions. The hormone stimulates the expression of chlorophyll-degrading enzymes, which liberate chlorophyll, reducing their levels for photosynthesis [59]. Increased ABA levels in rice upregulate the expression of various ABA-responsive genes, including those encoding dehydrins and late embryogenesis abundant proteins, which provide cellular protection, allowing the cell to withstand lethal stress [60].

In maize, ABA is essential for drought repression and photosynthesis adjustment. ABA-induced stomatal closure reduces stomatal conductance and transpiration rate, helping to maintain water deficit but at the same time restraining the rate of CO_2_ diffusion into the leaf, reducing photosynthesis. Additionally, ABA regulates chlorophyll degradation, with cells degrading light-absorbing molecules during the earlier stages of water deficit [61].

Ethylene is a gaseous signal molecule affecting drought repression and photosynthesis. Low ethylene levels induce adventitious root formation, promoting gas diffusion and water uptake due to increased root surface area. Simultaneously, high levels stimulate accelerated leaf senescence and acclimatization, leading to reduced productivity and photosynthetic ability of the plant. Ethylene plays a pivotal role in conjunction with ABA treatment. Furthermore, ethylene regulates chlorophyll content by controlling gene expression related to chlorophyll degradation and synthesis, helping light absorption and maintaining photosynthesis activity during water deficit [62]. In maize, ethylene influences chlorophyll content by regulating genes involved in chlorophyll degradation and synthesis, impacting light absorption and photosynthetic efficiency during drought stress [62].

Cytokinins are plant growth hormones that promote cell division. They play complex roles when the plant is subjected to water deficit. When the plant is subjected to low water conditions, cytokinin levels decrease, leading to reduced shoot growth and increased root development. Agurla et al. illustrated that when rice plant cells are subjected to water-deficient conditions, ABA levels increase, leading to stomatal closure [63]. Under drought conditions, rice plants have a higher root-to-shoot ratio, which is linked to the reduction of cytokinin levels. Cytokinins reduce the influence of ABA in roots and promotes shoot growth. Additionally, cytokinins play a vital role in delaying chlorophyll degradation and programmed cell death of leaves. In maize, reduced cytokinin levels under drought stress contribute to increased root growth and enhanced water uptake capacity. Cytokinins protect chlorophyll content and delay senescence, ensuring sustained photosynthetic activity and overall plant health [64].

### 4.5. Antioxidant Systems

Protection of photosystems from oxidative stress is a critical aspect of biological systems in plants, achieved through the use of antioxidant systems. Photosystems may be safeguarded from ROS damage by enzymatic and non-enzymatic antioxidants operating together to neutralize ROS (Figure 2).

Drought stress significantly impacts the relative water content (RWC) of plant tissues, a critical indicator of plant water status and its ability to maintain physiological functions under water-limited conditions [65,66,67]. The reduction in RWC during drought stress is primarily due to water loss through transpiration exceeding the plant’s ability to absorb water from the soil, leading to cellular dehydration, reduced cell turgor, and impaired metabolic activities, including photosynthesis.

Antioxidant enzymes play a pivotal role in modulating the effects of drought stress on RWC by mitigating oxidative damage and maintaining cellular homeostasis. Enzymatic antioxidants such as superoxide dismutase (SOD), catalase (CAT), and peroxidase (POD) are crucial in scavenging reactive oxygen species (ROS), thereby protecting cellular structures and functions. For instance, SOD catalyzes the dismutation of superoxide radicals into hydrogen peroxide, which is subsequently detoxified by CAT and POD into water and oxygen. This detoxification process helps maintain the integrity of cell membranes and prevent the loss of cellular water content [68]. Studies have shown that drought-resistant plant varieties exhibit higher activities of these antioxidant enzymes compared to susceptible ones, suggesting their role in enhancing drought resilience [69].

In rice, enzymatic antioxidants function effectively under drought with increased enzymatic activity. SOD, CAT, and POD, among others, participate in developing protective mechanisms for cellular components [9,70]. This process plays a critical role in reducing oxidative stress in the chloroplasts. This fact is supported by the observation that drought-resistant varieties exhibit higher activity of these antioxidants than susceptible ones [71]. The upregulation of genes encoding these enzymes, such as OsSOD and OsCAT, further supports their role in enhancing drought resilience [72].

Non-enzymatic antioxidants also contribute significantly to drought resistance in rice. Phenols, polyphenols, flavonoids, and anthocyanins contribute to ROS accumulation during drought. These compounds donate electrons to ROS to neutralize them. For instance, polyphenols quench singlet oxygen and peroxyl radicals to reduce lipid peroxidation [73]. Anthocyanins enhance photoprotection by absorbing excess light to reduce photo-oxidative stress [74].

In maize, enzymatic antioxidants also form the frontline of cellular protection. SOD, CAT, and ascorbate peroxidase (APX) activities are well pronounced when the maize plant is exposed to drought. Their increased activity in drought-resistant varieties is the evidence of their effectiveness in drought stress reduction. This is supported by the information that ZmSOD and ZmCAT in maize are highly regulated [75,76].

Drought triggers non-enzymatic antioxidants, including ascorbate (vitamin C) and other compounds that play a critical role in scavenging ROS in plants. Ascorbate participates in the ascorbate-glutathione cycle, directly detoxifying hydrogen peroxide and regenerating other antioxidants. Glutathione, a tripeptide, acts as a substrate for glutathione peroxidase and plays a critical role in maintaining redox homeostasis. Carotenoids, such as beta-carotene and lutein, play a significant role in reducing photoinhibition due to excess energy absorption, thus protecting the photosynthetic apparatus by quenching singlet oxygen and dissipating excess energy as heat [77].

PSII is subject to frequent damage, considering its vulnerability to oxidative damage due to its role in the light-dependent reactions of photosynthesis, where the splitting of water molecules generates oxygen and, consequently, ROS. The D1 protein of PSII, which is integral to the photosynthetic electron transport chain, is a frequent target of oxidative damage. The antioxidant defense systems help protect the D1 protein and other components of PSII, thereby preserving the efficiency of the photosynthetic apparatus [77]. Additionally, antioxidants play a role in maintaining the redox balance within chloroplasts, ensuring that the light-dependent and light-independent reactions of photosynthesis proceed efficiently.

PODs are critical enzymes in the plant’s response to drought stress. PODs catalyze the reduction of hydrogen peroxide (H_2_O_2_) to water and oxygen, a crucial step in the detoxification of ROS. Increased POD activity under drought conditions helps in maintaining RWC by reducing oxidative damage and preserving cellular integrity. This process not only protects the cell membrane but also ensures the retention of water within cells, thereby sustaining turgor pressure and overall plant health. Enhanced POD activity in drought-tolerant varieties of rice and maize correlates with improved RWC, highlighting its significance in drought response [78,79].

During the detoxification of ROS like hydrogen peroxide, the role of antioxidant en zymes is critical. SOD first converts superoxide radicals to hydrogen peroxide, which is then detoxified by CAT and POD into water and oxygen. The produced water (H_2_O) from these reactions is not just a byproduct but plays a vital role in maintaining cellular hydration and turgor pressure, especially under drought conditions. The transportation of this water within the plant is facilitated by aquaporins, membrane proteins that regulate water movement across cell membranes. Enhanced expression of aquaporins under drought stress aids in the efficient redistribution of water produced during ROS detoxification, thereby supporting cellular functions and improving overall plant water use efficiency [80].

Overall, the enhanced activity of enzymatic antioxidants like POD, SOD, CAT, and APX, along with the accumulation of non-enzymatic antioxidants such as polyphenols, anthocyanins, ascorbate, and glutathione, is crucial for mitigating oxidative stress and protecting photosystems in rice and maize under drought conditions. These antioxidant systems ensure the maintenance of photosynthetic efficiency and overall plant health, contributing to the drought tolerance of these important crop species. Understanding these mechanisms provides valuable insights for developing drought-resistant crops and improving agricultural sustainability.

### 4.6. Leaf Characteristics

Drought stress causes a variety of changes in plant leaf morphology and anatomy to prevent water wastage and sustain photosynthetic performance (Figure 2). These include reduced leaf area, increased leaf thickness, alterations in stomatal traits, and the shrinking of bulliform cells. Characterizing these changes in the leaves of drought-sensitive species is key to understanding the role of these adaptations in drought tolerance. The stomatal crypts and reduced stomatal density of both rice and maize plants lower water loss via transpiration.

Reduced leaf area is a key drought response in plants. By forming small leaves, leaf reduction lowers the overall surface area through which transpiration occurs, effectively conserving water. Plants may produce smaller individual leaves or fewer leaves per plant to reduce leaf area. Hormonal regulation controls the reduction in rice leaf area, where increased ABA concentrations inhibit leaf expansion and promote senescence [81]. Similarly, ABA levels are upregulated in maize, reducing leaf area and helping balance water availability with water uptake for drought tolerance [82]. The drought resistance mechanisms are supported by the expression of drought-responsive genes that regulate leaf growth and development.

Increased leaf thickness is another vital anatomical adaptation to drought stress. Thicker leaves usually host more mesophyll cells than thin leaves, allowing them to store more water and maintain turgor pressure when the plants are exposed to drought. This increased thickness also enhances the leaf’s structural integrity, making it more resistant to wilting and mechanical damage. Drought induces leaf thickness development in rice, increasing the leaf’s ability to access light and shielding the photosynthetic machinery from direct light exposure [83]. Similarly, maize exhibits thicker leaves under drought conditions, a feature partially caused by a well-developed palisade mesophyll layer that improves light capture efficiency even when stomata are partially closed [84].

Lipid dynamics also play a crucial role in plant response to drought stress, impacting both the cuticular barrier and internal cellular membranes. The cuticle, composed of cutin and cuticular waxes, is a vital protective layer on the leaf surface that prevents excessive water loss. Drought conditions can lead to significant changes in the composition and structure of these lipid components, enhancing the plant’s ability to retain water. The plant cuticle is an extracellular hydrophobic layer that covers the aerial parts of the plant, primarily consisting of cutin and cuticular waxes. Cutin is a polymer matrix made up of hydroxylated fatty acids and glycerol, while cuticular waxes are complex mixtures of very long-chain fatty acids (VLCFAs), alkanes, aldehydes, ketones, alcohols, and esters. These components work together to form a barrier against water loss and protect against environmental stresses [85]. In drought conditions, plants often enhance the production and deposition of cuticular waxes to reduce water permeability. Studies on maize (*Zea mays*) have shown that increased wax deposition can significantly improve drought tolerance by reducing cuticular conductance and transpiration rates [86]. Similarly, rice (*Oryza sativa*) plants with thicker cuticles and higher wax content exhibit better water retention under drought stress [87].

Lipid remodeling within cellular membranes is another critical response to drought stress. This process involves the reorganization of membrane lipids to maintain cell integrity and function during dehydration. Drought stress induces the accumulation of specific lipid species, such as phospholipids and galactolipids, which stabilize membrane structures and prevent leakage [88]. For example, phosphatidylcholine and phosphatidylethanolamine levels increase to maintain membrane fluidity and functionality under low water conditions [88]. Additionally, the remodeling of thylakoid membranes in chloroplasts is essential for maintaining photosynthetic efficiency during drought. The enrichment of specific galactolipids, such as monogalactosyldiacylglycerol (MGDG) and digalactosyldiacylglycerol (DGDG), helps protect the photosynthetic apparatus from oxidative damage and ensures the proper functioning of photosystems [89,90].

Alterations in stomatal density and distribution are critical for enhancing drought tolerance. Stomata are responsible for gas exchange, including CO_2_ uptake for photosynthesis and water loss through transpiration. Their size and number are thus modified under drought conditions to balance gas exchange and water conservation. In rice, reduced stomatal density helps limit transpiration while maintaining sufficient CO_2_ uptake for photosynthesis [91]. Additionally, the development of sunken stomata or stomatal crypts in rice can further reduce transpiration rates by protecting the stomata from direct exposure to air currents [92].

Bulliform cells, also known as motor cells, play a crucial role in drought tolerance by facilitating leaf rolling and unrolling [83]. These large, bubble-shaped epidermal cells are located on the upper surface of grass leaves, including those of rice and maize. Under drought conditions, bulliform cells lose turgor pressure, causing the leaves to roll up and reduce their exposed surface area, thus minimizing water loss through transpiration. This leaf rolling mechanism is an effective drought avoidance strategy that helps maintain leaf hydration and protects the photosynthetic machinery from excessive light exposure and photo-oxidative damage. In rice, the degree of leaf rolling is correlated with drought tolerance, with more pronounced leaf rolling observed in drought-tolerant varieties [93]. Similarly, maize plants exhibit leaf rolling as a drought response, which helps conserve water and sustain photosynthetic activity during periods of water deficit.

### 4.7. Improvement of Water Use Efficiency

The other critical strategy plants use for survival and growth in water-deficient conditions is through improved water use efficiency (WUE) (Figure 2). Plants employ various strategies to improve WUE, which in turn helps maintain higher RWC under drought conditions. WUE refers to the ratio of the total biomass produced by a plant to the amount of water it uses, and it measures a plant’s ability to efficiently utilize water to produce the necessary energy and nutrients for growth and development. Plants use several tactics to increase WUE by controlling stomata and changing leaf structure to maintain higher RWC under drought conditions.

Stomatal regulation plays a vital role in balancing water conservation, CO_2_ uptake, and maintaining RWC, essential for photosynthesis. This process is finely tuned in drought-tolerant varieties, which exhibit enhanced ABA sensitivity, optimizing the balance between water conservation and photosynthesis [94]. In rice, drought stress induces stomatal closure mediated by the plant hormone ABA. ABA levels increase under drought conditions, promoting stomatal closure to reduce transpiration and conserve water [59]. This response must be finely tuned to maintain sufficient CO_2_ intake for photosynthesis. Drought-tolerant rice varieties often exhibit enhanced ABA sensitivity, allowing them to optimize this balance more effectively, thereby improving WUE. In maize, a C_4_ plant, stomatal regulation is similarly crucial. Maize plants exhibit a higher intrinsic WUE due to their C_4_ photosynthetic pathway, which concentrates CO_2_ at the site of Rubisco, reducing photorespiration and enhancing photosynthetic efficiency even when stomata are partially closed [95]. This mechanism allows maize to sustain higher photosynthetic rates and better WUE under drought conditions, contributing to the maintenance of RWC [95].

Anatomical changes in leaves also play a significant role in improving WUE and maintaining RWC. Rice plants often develop thicker leaves with more mesophyll cells, which can store more water and maintain turgor pressure during drought. Reduced leaf area is another adaptation that minimizes water loss through transpiration. Changes in stomatal density and distribution, such as the development of sunken stomata, further reduce water loss while ensuring adequate CO_2_ uptake for photosynthesis [83,92]. In maize, leaf rolling is a common drought response that reduces leaf area exposed to sunlight and air, thereby lowering transpiration rates. This morphological adaptation, along with a higher density of mesophyll cells, helps maintain photosynthetic activity under water-limited conditions [96].

Overall, maintaining higher RWC under drought conditions is closely linked to improved WUE. By regulating stomatal closure and adapting leaf anatomy, plants like rice and maize can conserve water, sustain metabolic activities, and ensure better growth and productivity under drought stress. Understanding these mechanisms provides valuable insights for developing drought-resistant crops and improving agricultural sustainability [97,98].

### 4.8. Root Structure Regulation

Root structure regulation is another critical adaptation mechanism by which plants increase water uptake under drought (Figure 2). Water uptake maintenance through root system modifications, which includes alterations in root architecture with deep root system development, reduced lateral root branching density and root-to-shoot ratio increase, is an essential aspect of plant survival under drought stress [99]. This mechanism is well exemplified by various crop species, such as rice, maize, sorghum, and soybean, demonstrating distinct strategies they use to cope with drought stress.

Many crop species, in general, utilize root depth development as the main response mechanism to drought conditions. For example, rice plants possess a relatively shallow root system under watered conditions, rendering them susceptible to drought. However, under low moisture conditions, rice plants extend their roots into deeper soil layers to access the soil moisture. This process is triggered by hormonal signaling, mainly abscisic acid, which elongates primary roots while inhibiting lateral branching. Additionally, plant response to drought conditions upregulates the *DRO1* (*DEEPER ROOTING1*) gene, a deeper rooting phenotype factor, increasing root growth angle and penetration capability [100].

Maize displays a similar response to drought through developmental changes in root depth and an increase in root mass in deeper soil layers [101]. Rooting involves the crown and seminal roots, with genes triggering and controlling root initiation and elongation processes. Plastic response of root in the form of reduced lateral production and branching density has been shown to improve the drought tolerance of maize plants under greenhouse and field conditions [102,103]. The plant root-to-shoot ratio is also increased due to enhanced root growth at the expense of shoot development. Furthermore, plant transpiration is diminished due to the reduced leaf area, enhancing overall water-use efficiency [104]. In addition, more lignin deposition on cell walls in root vascular bundle tissues (VBT) under drought stress to forming a water-resistant barrier around mature xylem tissues [4].

Sorghum, in turn, is characterized by extensive root system development throughout soil layers. This plant accumulates osmolytes, such as proline and glycine betaine, that maintain root cell turgor pressure, ensuring root function in deep conditions. The root-to-shoot ratio is increased in sorghum due to enhanced root growth at the expense of shoot growth [105,106].

Soybean increases root depth and branching under drought through hormonal triggering, which includes increased ABA and decreased cytokinins. Furthermore, drought-responsive gene upregulation, such as *GmNAC* and *GmDREB*, enhances root development processes in soybean [107,108].

## 5. Conclusions and Future Directions

Drought stress significantly impacts crop yield by affecting various physiological and reproductive processes in plants. Yield reduction under drought conditions is often due to impaired photosynthesis, reduced nutrient uptake, and compromised reproductive development. Plants employ several strategies to maintain yield under drought stress, which can be considered adaptive mechanisms to enhance drought resilience.

The pervasive impact of drought on photosynthesis in major food crops underscores the urgent need for understanding and mitigating this environmental stress. Drought stress adversely affects both the light-dependent and light-independent reactions of photosynthesis, leading to significant reductions in crop yield and quality. The disruption of photosynthetic processes is primarily due to impaired chlorophyll function, reduced CO_2_ availability from stomatal closure, and oxidative damage to cellular structures. These effects are evident in crops, which are vital for global food security.

To mitigate the adverse effects of drought, plants have evolved various adaptive mechanisms involving both structural and physiological changes. NPQ mechanisms and alternative electron transfer pathways, such as CET and chloroplast respiration, are critical for maintaining photosynthetic efficiency under drought conditions [52,54,55,109]. Enhanced antioxidant systems, including enzymatic antioxidants like CAT and POD, as well as non-enzymatic antioxidants such as polyphenols, flavonoids, and anthocyanins, play a crucial role in protecting the photosynthetic apparatus from oxidative stress [68,73,74]. Hormonal regulation, particularly by ABA, ethylene, and cytokinins, plays a central role in modulating stomatal closure, root growth, and stress responses, optimizing plant growth and photosynthetic efficiency under drought stress [59,64,110]. Structural adaptations in leaves, such as increased thickness, reduced area, and altered stomatal density, help conserve water while maintaining CO_2_ uptake for photosynthesis [83,92]. Root adaptations, including deeper root systems and increased root-to-shoot ratios, enhance water uptake from deeper soil layers, crucial for accessing water in arid environments [93].

Future research in this direction should focus on identifying and integrating drought-tolerant traits into crop breeding programs. Genetic engineering and molecular breeding techniques offer promising avenues for developing crop varieties with enhanced drought tolerance. Understanding the molecular mechanisms underlying drought responses, particularly those related to photosynthesis and chloroplast function, will be crucial for these efforts. Moreover, exploring the roles of plant hormones, such as ABA, ethylene, and cytokinins, in modulating drought responses can provide insights into developing crops with optimized stress resilience.

Agronomic practices also play a pivotal role in mitigating drought impacts. Efficient water management strategies, including the use of drought-tolerant crop varieties, improved irrigation techniques, and soil moisture conservation practices, are essential for sustainable agriculture. Integrating these practices with advanced breeding strategies will enhance the resilience of agricultural systems to drought stress, ensuring food security in the face of climate change and increasing resource demands.

In conclusion, addressing the challenges posed by drought stress requires a multidisciplinary approach that combines genetic, physiological, and agronomic strategies. By enhancing our understanding of the complex interactions between drought stress and photosynthetic processes, and by developing innovative solutions, we can improve the resilience of major food crops, thereby safeguarding global food security. Continued research and collaboration among scientists, breeders, and farmers will be essential for developing and implementing effective strategies to combat the adverse effects of drought on agriculture.

## Figures and Tables

**Table 1:** Major crops yield statistics by country (region) in 2023/2024.

Country	Major Crops	Area (1000 Ha)	Yield (T/Ha)
China	*Zea mays* (maize)	44,218	6.5
	*Oryza sativa* (rice)	28,949	7.1
	*Triticum aestivum* (wheat)	23,627	5.8
	*Glycine max* (soybean)	10,470	2.0
	*Arachis hypogaea* (peanut)	4820	3.9
USA	*Zea mays* (maize)	35,011	11.1
	*Glycine max* (soybean)	33,328	3.4
	*Triticum aestivum* (wheat)	15,084	3.3
	*Sorghum bicolor* (sorghum)	2475	3.3
	*Oryza sativa* (rice)	1155	8.6
India	*Oryza sativa* (rice)	48,000	4.2
	*Triticum aestivum* (wheat)	31,401	3.5
	*Zea mays* (maize)	11,000	3.4
	*Panicum miliaceum* (millet)	9500	1.4
	*Glycine max* (soybean)	13,000	0.9
Brazil	*Glycine max* (soybean)	45,900	3.4
	*Zea mays* (maize)	21,500	5.7
	*Triticum aestivum* (wheat)	3470	2.3
	*Oryza sativa* (rice)	1545	6.9
	*Sorghum bicolor* (sorghum)	1541	3.1
EU	*Triticum aestivum* (wheat)	24,200	5.5
	*Zea mays* (maize)	8280	7.4
	*Hordeum vulgare* (barley)	10,300	4.6
	*Brassica napus* (rapeseed)	6220	3.2
	*Helianthus annuus* (Sunflower seed)	4801	2.1

The top five major crops in each country (region), listed in order of production in tons, are shown with their cultivation area (in thousand hectares) and yield (tons per hectare). USA: The United States of America. EU: The European Union. Data are sourced from https://ipad.fas.usda.gov/countrysummary (accessed on 29 May 2024).

## Data Availability

No new data were created or analyzed in this study. Data sharing is not applicable to this article.
